# A New Wearable System for Personal Air Pollution Exposure Estimation: Pilot Observational Study

**DOI:** 10.2196/60426

**Published:** 2025-07-04

**Authors:** Sara Bernasconi, Alessandra Angelucci, Andrea Rossi, Andrea Aliverti

**Affiliations:** 1 Department of Electronics, Information and Bioengineering, Politecnico di Milano, 32 Piazza Leonardo Da Vinci, Milan, 20133, Italy, 39 3451728554

**Keywords:** air quality, body sensor network, personal exposure, cardiorespiratory monitoring, wearable system

## Abstract

**Background:**

Air pollution is a major environmental cause of premature deaths, responsible for around 7 million deaths annually. In this context, personal air pollution exposure (PAPE), the product of pollutant concentration and minute ventilation (*V’m*), is a crucial measure for understanding individual health risks. Standard exposure techniques do not address the space-time variability of air pollution, both indoor and outdoor, and the intra- and intersubject variability in *V’m*.

**Objective:**

This study evaluates the feasibility of using a wearable body sensor network (BSN) to estimate PAPE in real-life settings, assess its capability to detect spatiotemporal variations in pollution levels, and compare inhaled dose estimates from the BSN with those from fixed monitoring stations and standard *V’m* values. The study also examines the system’s usability.

**Methods:**

The system, a BSN capturing physiological (pulse rate [PR] and respiratory rate [RR]) and environmental data, including health-affecting pollutants (particulate matter [PM] 1, PM2.5, PM10, CO_2_, CO, total volatile organic compounds, and NO_2_), was tested in a 4.5 km walk in Milan by 20 healthy volunteers. PR and RR collected by the system were used, together with biometric data and forced vital capacity estimations, in a model for *V’m* estimation to compute PAPE. Pollution levels were compared between morning and afternoon measurements, as well as between indoor and outdoor settings.

**Results:**

Variations in RR were found among volunteers and at different locations for the same participant. Significant differences (*P*<.001) in pollutant concentrations were observed between morning and afternoon for CO_2_ (higher in the morning) and PM (higher in the afternoon). Spatial variability along the walking path was also detected, highlighting the system’s high spatiotemporal resolution. Indoor environments showed high variability in CO_2_ and total volatile organic compounds, while outdoor settings exhibited elevated and variable PM levels. The mean PAPE to PM2.5 estimated with tabulated *V’m* and fixed station data was 13.31 (SD 4.16) μg while the one estimated with the BSN was 16.27 (SD 9.78) μg, 2.96 μg higher (22.3%; 95% CI −6.55 to 0.63; *P*=.05) than the former one, and with a broader IQR. Nevertheless, the 2 estimation methods show a good and strongly significant correlation (*r*=0.665; *P*<.001). The system’s usability was generally rated as good.

**Conclusions:**

The BSN provides high-resolution spatiotemporal data on personal exposure, capturing differences in pollution levels dependent on time, location, and surrounding environment, along with individual physiological variations. It offers a more accurate estimation of inhaled dose in real-life settings, supporting personalized exposure assessments and potential applications in activity planning and complementing epidemiological research.

## Introduction

Air pollution is the main environmental trigger for premature deaths worldwide. A total of 9 out of 10 people breathe highly polluted air, as stated by the Organization for Economic Co-operation and Development Outdoor and household air pollution causes around 7 million deaths every year, 90% of which occurs in low- and middle-income countries because of open fires and stoves used for cooking purposes [1].

Reducing environmental pollution is a widespread concern and the United Nations has included it among the Sustainable Development Goals, more specifically in relation to the third goal about good health and well-being.

In this alarming scenario, accurate monitoring of the air we breathe is fundamental, both from an individual point of view, as a way of enhancing awareness and promoting a healthy lifestyle, and from a collective point of view, to foster new public policy oriented towards improving air quality. For instance, in the context of public intervention, monitoring and control over air pollution are among the foundation stones of smart cities [2,3].

Nowadays, there are various technologies for monitoring air pollution. Fixed stations are located in various geographical areas to assess the environment, which in turn is characterized by different predominant emission sources and different distribution and density of buildings. In addition, in some countries, such as Italy, mobile stations are used for a predetermined period (eg, 2 weeks) to investigate pollution variability during the year and examine specific situations, following notification by the municipal administrations [4]. Moreover, satellite observations help identify large areas of pollution. For instance, the Sentinel-5 Precursor satellite from the European Space Agency produces one image per day for different pollutants. These measurements are crucial for protecting public health, preserving the environment, guiding policy decisions, mitigating climate change, and promoting sustainable development, but operate at a macro-geographical and population level and give little insight into more specific data at the individual level.

Generally, studies conducted at the individual level approximate a person’s level of exposure to various pollutants to the outdoor pollution level at their residential address. As demonstrated by Lu [5], ignoring human motion and spatiotemporal variability of air pollution could lead to differential exposure misclassification, potentially biasing health risk assessments. This happens because standard monitoring techniques are not sufficient to account for the great space-time variability of pollution [6]. Indeed, air quality can drastically change over short distances, lower than 100 m [7], and short time intervals [8] due to its pronounced dependence on both pollution sources and air circulation. As an example, “urban street canyons”, that is, streets lined with tall buildings that impede air circulation, can give rise to a pollution scenario that can be completely different from the surrounding one.

As mentioned before, indoor air quality must be monitored as well, as demonstrated in multiple research studies [9,10]. In fact, people spend between 80% and 90% of their time in closed environments and the World Health Organization reports 1.6 million premature deaths caused by indoor air pollution [11].

However, focusing solely on indoor exposure results in a significant underestimation of exposure levels [5]. For example, particulate matter [PM] 2.5 exposure measured exclusively in indoor environments is estimated to be 61% lower than actual exposure for commuting distances exceeding 30 km. For these reasons, a personal air quality monitor is suggested to accurately assess exposure to pollutants, taking into account the participant’s mobility between indoor and outdoor environments [12].

Various companies have developed portable systems capable of monitoring key air pollutants [6]. Among them, it is worth mentioning the Atmotube PRO (Atmotech Inc), which provides continuous monitoring of PM and volatile organic compounds (VOCs), and Flow (Plume Labs) which measures PM, VOCs, and nitrogen dioxide (NO_2_). Further examples include the Wynd Air Quality Tracker (WYND), which specializes in PM monitoring, and Aeroqual’s portable air quality monitor (Aeroqual), which supports the integration of different sensors in a limited configuration.

Nevertheless, for an accurate assessment of the pollution that effectively infiltrates human organisms, the respiratory activity of the participant assumes a crucial role. To consider the whole phenomenon, personal air pollution exposure (PAPE) is the appropriate measure, as proposed by Arano et al [8]. PAPE is evaluated considering a respiratory factor and an environmental factor. It is calculated with the following formula:


(1) $$PAPE\left(p\right)=SZ\left(p\right)*V\prime_{m}$$

Where $V\prime_{m}$ is minute ventilation in m^3^/min and $SZ\left(p\right)$ is the fully aggregated pollution value in a certain period *p,* calculated as:


(2) $$SZ\left(p\right)=\sum_{t=1}^{n}\frac{1}{2}(Z_{t_{i+1}}+Z_{t_{i}})(t_{i+1}-t_{i})$$

Where $Z_{t_{i+1}}$ and $Z_{t_{i}}$ are the specific pollutant concentrations in the time instants of $t_{i+1}$ and $t_{i}$, with $t_{i+1}-t_{i}$ should be lower than 10 minutes.

Hence, a wearable system following the person everywhere, monitoring both indoor and outdoor environments and the cardio-respiratory activity of the person, can represent a robust solution for the accurate assessment of PAPE. In this study, we have carefully structured our experimental design to systematically address multiple research questions. Our primary objective is to evaluate the feasibility of using a body sensor network (BSN) of wearable devices to estimate personal pollutant exposure in real-life settings. To validate the performance of the BSN, we compare the inhaled dose [13] calculated from the system’s measurements with the dose derived from fixed monitoring station data and tabulated $V_{m}^{\prime }$ values. Furthermore, we assess the system’s ability to detect variations in pollution levels across different times and locations. Additionally, we seek to determine the usability of the BSN in both indoor and outdoor environments. These distinct yet interconnected objectives are central to advancing our understanding of the system’s potential applications and limitations. In section II, the novel system and the experimental protocol involving 20 healthy participants are detailed. Results about pollutant distributions and evaluation of PAPE to air pollutants are reported and discussed in section III. In section IV conclusions are drawn, highlighting limitations and future developments.

## Methods

### Environmental Monitor and Cardio-Pulmonary Monitoring Platform

**Figure 1.**
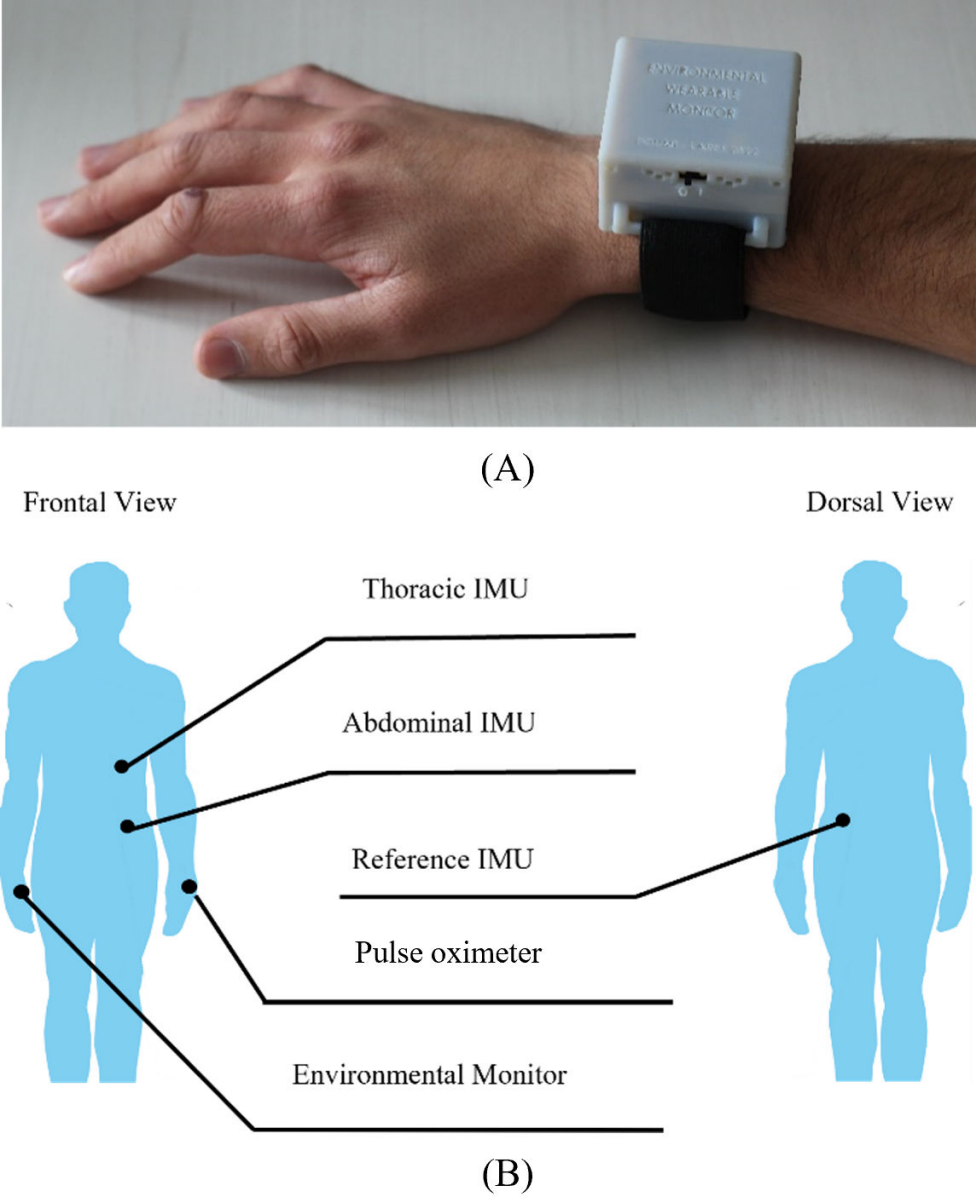
(A) Wearable device for environmental monitoring; (B) Body sensor network composed of 3 IMUs, a wrist-worn pulse oximeter, and a wrist-worn environmental monitor. IMU: inertial measurement unit.

The developed wearable environmental monitor, shown in Figure 1A, is designed to be worn on the wrist or on the arm to track the pollutant concentrations to which the participant is exposed [14].

The monitored pollutants are PM (specifically PM1, PM2.5, and PM10; where the number indicates the diameter of the particle in µm), CO_2_, total volatile organic compounds (tVOCs), carbon monoxide (CO), and NO_2_. The PM sensor is the SPS30 by Sensirion (accuracy of ±10 μg/m^3^ for PM1 and PM2.5, ±25 μg/m^3^ for PM10) [15], the CO sensor is the SCD41 (±40 ppm of accuracy) [16], the tVOC sensor is the SGP30 (1.3% of measured value with Ethanol and H_2_) [17], and the CO and NO_2_ sensor is the MICS-6814 by SGX [18]. Atmospheric parameters such as temperature, humidity, and pressure are also detected using the Bosch BME280 sensor. The environmental monitor node is integrated into a BSN, shown in Figure 1B, aimed at collecting cardio-respiratory data. The BSN is composed of 3 devices each embedding an inertial measurement unit (IMU), positioned one on the thorax, one on the abdomen, and a reference one on the back [19-21], and a wrist-worn pulse oximeter [22], in addition to the environmental monitor.

During the experimental campaign, data recorded by all the nodes of the BSN were exploited to evaluate the PAPE. The sample frequency of the 3 IMUs and the pulse oximeter is 10 Hz for each of them and data are sent through the ANT communication protocol in a shared channel network configuration. Instead, the environmental monitor has a sampling frequency of 0.042 Hz and sends a new set of data every 24 s in an independent channel for data transmission to the master which, in our case, is a smartphone. An Android application displays environmental data in real time and geolocates them using the integrated smartphone GPS. Both environmental and physiological data are saved in a cloud-hosted database (ie, Google Firebase) for later analysis.

### Inclusion and Exclusion Criteria

Volunteers were eligible to participate if they were between 18 and 75 years of age. However, individuals were excluded if they exhibited any physical deformities in the upper limbs that might compromise the validity of the data obtained. Anyone with gait impairments likely to interfere with the ability to complete the study protocol was also excluded.

### Experimental Campaign

**Figure 2.**
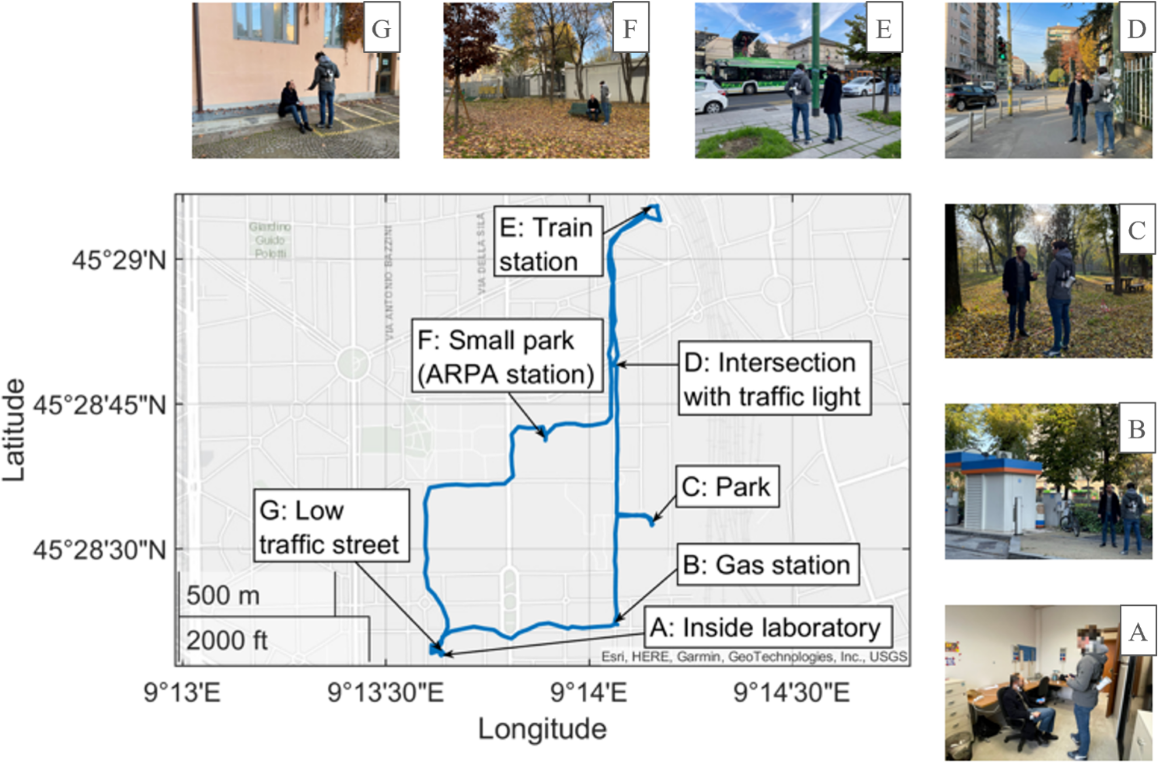
Route of the experimental campaign with static acquisition points highlighted. They are (A) inside a laboratory, (B) near a gas station, (C) in a park, (D) at an intersection with traffic lights, (E) near a train station, (F) in a small park where there is also an ARPA station, and (G) in a low traffic street. ARPA: Agenzia Regionale per la Protezione Ambientale.

The experimental protocol consisted of a walk over 4.5 km in the university area of Politecnico di Milano, passing through some specific places. The full path is reported in Figure 2. Two different types of acquisition were performed as follows: static and dynamic. Static acquisitions consisted of 5 minutes of recording of the overall system in a static condition in 7 acquisition points. Each point represents a distinct microenvironment: a laboratory (A), a gas station (B), a park (C), an intersection with traffic light (D), a train station (E), a small park (F) in which there is also a fixed station of the Lombardy regional agency for environmental protection (Agenzia Regionale per la Protezione Ambientale; ARPA) and a low traffic street (G). Participants were seated in points A, F, and G, and standing in points B, C, D, and E. Dynamic acquisitions involved only the environmental monitor and consisted of the recording of pollutant concentration while walking from one static point to the other. Most acquisitions were performed in November 2022. Two time slots per day were available, one in the morning around 10:00 AM and one in the afternoon around 2:30 PM. This choice was made to avoid peak traffic hours and keep as much as possible the same conditions during all the acquisitions.

### Preprocessing and Normalization of Environmental Data for Comparative Analysis

Environmental data were resampled with a sampling frequency of 1 Hz. Then, the entire acquisition was segmented using time stamps registered during the acquisition protocol when arriving at and leaving each acquisition point. This process generates 7 arrays for static conditions and 7 arrays for dynamic acquisition for each participant, serving as a means of space normalization to facilitate the comparison of acquisitions across different volunteers. In fact, given the hypothesis of variability in pacing among volunteers and that an uncontrolled environment is susceptible to random events (eg, red traffic lights or crowds affecting walking speed), this approach ensures a more consistent basis for comparison.

A linear interpolation that takes 3 samples per minute is applied to obtain the same number of samples for each participant in each segmented array.

After these processing steps, different pollutants and their variation along the path were plotted and visually inspected. Considering that ARPA provides a single daily value for PM2.5 and PM10, our comparison with data recorded by the fixed station (F) focused on acquisitions conducted in the morning and afternoon of the same day.

### Personal Air Pollution Exposure

The assessment of the inhaled dose, or PAPE, involved the use of Equation (1) and was computed for PM1, PM2.5, PM10, tVOC, and CO_2_. The minute ventilation in (1) is computed as the product of the respiratory rate (RR) and tidal volume. Since tidal volume information are not provided by the system, a model proposed by Greenwald et al [23] was instead deployed for the respiratory factor. This model is in the form of a power function, reported in (3), based on a large, pooled dataset, with a median percent error of 1.20% (IQR 37.9%).


(3) $$V\prime_{m}=e^{-8.75}HR^{1.72}RR^{0.611}age^{0.298}sex^{-0.206}FVC^{0.614}$$

Where $V\prime_{m}$ is the value of minute ventilation expressed in L/min, HR is the heart rate in beats per minute, and RR is the respiratory rate in breaths per minute, and FVC is the forced vital capacity in L. Pulse rate (PR) data are recorded by the pulse oximeter and are used instead of HR, whereas the 3 IMUs monitor RR. In this paper, FVC was evaluated using the Global Lung Function Initiative method from the European Respiratory Society [24]. The remaining variable is sex (1 for men, 2 for women).

For what concerns the environmental factor, it consists of a mean of the values acquired during 1 minute of measurement for all 5 minutes in the different static acquisition points. PAPE to PM2.5, assessed using data obtained from wearable sensors, was compared with what is hereafter referred to as the “Standard Method.” This method estimates PAPE based on tabulated values for $V\prime_{m}$, and daily pollutant concentration data from ARPA fixed monitoring stations. Specifically, in the Standard Method, $V\prime_{m}$ values were derived from tables considering age range, sex, and activity level [25].

In our study, we opted to align with an activity level of 3, characterized by METs typically falling within the range of 2 to 4, as an approximation to our experimental conditions. In fact, the trial encompassed brisk walking, an activity associated with a MET value of 5, and standing and seated positions, corresponding to MET values of 1.8 and 1.3, respectively. The tabulated minute ventilation for an activity level of 3 is 13.26 L/min for women and 15.14 L/min for men. These values were used as respiratory factors in the Standard Method.

### Questionnaire

At the end of the experimental protocol, a questionnaire, reported in Table 1, was verbally administered by the principal investigator or his collaborators to the participants to assess satisfaction with device usability. Answers were recorded, transcribed, and then the audio recordings were deleted. The recording was convenient as it allowed the participants to walk while answering, ensuring that the process did not interrupt the experimental activities and reducing the overall time required for the protocol.

**Table 1.** Items of the wearability questionnaire.

Item number	Item	Ideal scores
I1	I think the device is simple to wear	5
I2	I think I would be able to use the device autonomously	5
I3	I found the fixing method uncomfortable	1
I4	I would have preferred to remove the device during the test	1
I5	I think I could wear the device for a long time	5
I6	I think using the device would negatively affect my daily activities	1
I7	I think I would be able to use the smartphone app autonomously	5
I8	I think the data shown are easy to read	5
I9	I think the device has a functional design	5

Participants were asked to give a score between 1 (completely disagree) and 5 (completely agree) to the 9 statements. The collected responses were compared with the ideal scores (reported in Table 1) to evaluate satisfaction with the device, and the results were visualized using a radar plot.

### Statistical Analysis

First, the statistical difference in pollutants' concentration and physiological parameters between the 7 static acquisition points was assessed. After checking the normality of the distributions through the Shapiro-Wilk test, a Friedman test was performed. If the null hypothesis was rejected, a post-hoc test was performed to assess between which acquisition points there was a statistical difference in pollutants’ concentration and physiological parameters and the entity of this difference.

Then, concentrations of the morning and afternoon acquisitions of the same day were compared by means of a Wilcoxon signed rank test, aiming to assess potential statistical differences between these time intervals.

RR of the different participants was analyzed using Kruskal-Wallis one-way ANOVA on Ranks to determine the presence of any statistical differences. This assessment aimed to gauge whether a standard value of RR could effectively serve as a proxy for the actual real-time RR recorded during the protocol.

### Ethical Considerations

The experimental protocol was approved by Politecnico di Milano Ethics Committee (approval number 39/2022; date of approval October 10, 2022) and involved 20 healthy volunteers, who gave their informed consent. Participants retained the right to withdraw from the study protocol at any time, as outlined in the informed consent. Participant data were safeguarded through a process of pseudonymization, whereby each participant was assigned a unique identifier, with the key linking identifiers to individual identities securely maintained by the principal investigator. Participants were recruited through word of mouth and did not receive any financial compensation for their participation.

## Results

### Participants

The population consisted of 14 male and 6 female participants. The median of the age distribution was 25.5 years with an IQR of 3 years, with 3 outliers at 32, 46, and 55 years. The median body weight was 70 (IQR 67.5-76.5) kg and the median height was 179 (IQR 168.0-180.5) cm.

### Pollutant Concentration Trends: Indoor Versus Outdoor and Temporal Comparisons

**Figure 3.**
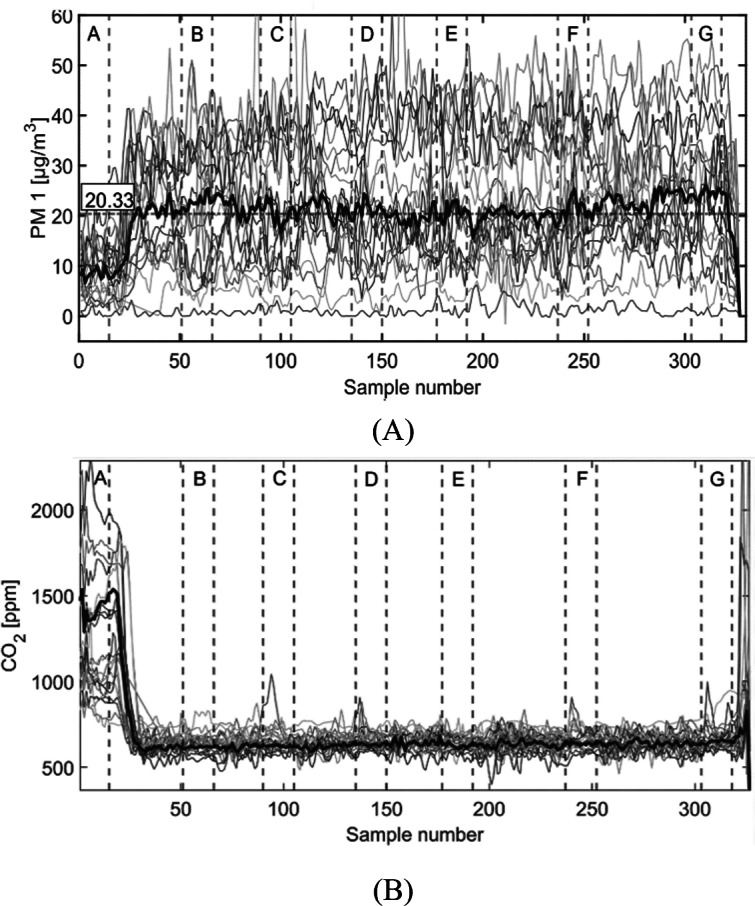
PM1 (A) and CO_2_ (B) concentration along the route for the 20 participants. The median is represented by the bold line. The vertical dashed lines show the 7 specific acquisition points, denoted by capital letters A through G: point A, inside a laboratory; point B, near a gas station; point C, in a park; point D, at an intersection with traffic lights; point E, near a train station; point F, in a small park where there is also an ARPA station; and point G, in a low traffic street. ARPA: Agenzia Regionale per la Protezione Ambientale; PM: particulate matter.

In Figure 3A and B, PM1 and CO_2_ concentrations, respectively, of the whole acquisitions are reported, with the median curve represented by the bold line. As expected, there is a strong variation moving from the indoor environment (acquisition point A, inside a laboratory) to the outdoor environment (all other acquisition points). Specifically, PM1 exhibits a significant increase outdoors, whereas CO_2_ shows the opposite trend. Inside the laboratory, significant variability and numerous high peaks of CO_2_ can be observed.

**Figure 4.**
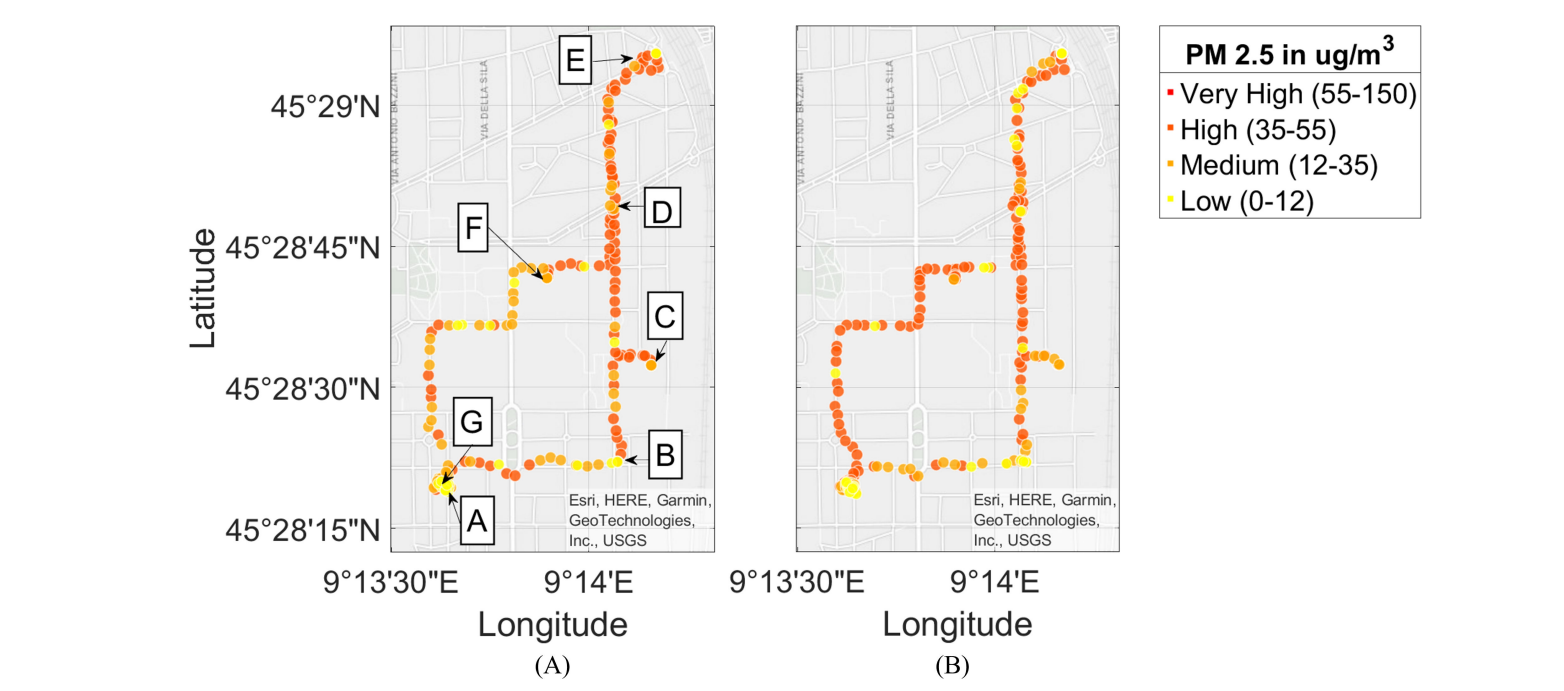
Map of the PM2.5 concentration during (A) morning acquisition and (B) afternoon acquisition of 2 participants, tested the same day. The 7 specific acquisition points are denoted by capital letters A through G: point A, inside a laboratory; point B, near a gas station; point C, in a park; point D, at an intersection with traffic lights; point E, near a train station; point F, in a small park where there is also an ARPA station; and point G, in a low traffic street. ARPA: Agenzia Regionale per la Protezione Ambientale; PM: particulate matter

Substantial variability in PM was observed for the outdoor segment of the acquisition path. This variability is evident in Figure 4, which illustrates PM2.5 concentrations for 2 participants tested on the same day during the morning (Figure 4A) and the afternoon (Figure 4B). For instance, it can be noted that along the same street, from acquisition point B to E, concentrations span from very low (0‐12 μg/m^3^) to very high (55‐150 μg/m^3^) values. Moreover, Figure 4 also shows a generally lower concentration of PM2.5 in the morning with respect to the afternoon for a representative day. All these considerations are true also for PM1 and PM10.

**Figure 5.**
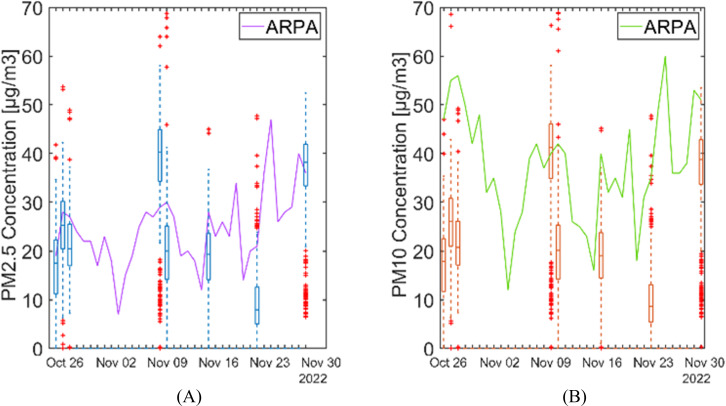
PM2.5 (A) and PM10 (B) concentrations’ comparison between ARPA values taken from the fixed station in point F (continuous line) and data recorded by our device when 2 participants were tested on the same day (boxes). ARPA: Agenzia Regionale per la Protezione Ambientale; PM: particulate matter.

In Figure 5, boxplots of the new device measurements and a solid line for ARPA values are reported. It can be observed that concentrations captured by the new device are often lower than the mean value of the day reported by ARPA.

NO_2_ and CO levels never overcame the detection limit of 20 ppb during our acquisitions.

### Quantitative Analysis of Pollutant Concentration and Physiological Parameters

For each pollutant, the Friedman test showed a statistical difference between acquisition points. In fact, for each gas and particulate, there was a statistical difference between the indoor environment and the outdoor environment. A higher value of CO_2_ and tVOCs was found inside the laboratory with respect to all other outdoor acquisitions. Instead, PM was higher outdoors and lower indoor. Medians and IQRs for each pollutant together with RR and PR in the different acquisition points are reported in Table 2.

**Table 2.** Concentrations of CO_2_, total volatile organic compounds (tVOCs), particulate matter (PM) 1, PM2.5, PM10, respiratory rate (RR), and pulse rate (PR) in the 7 specific acquisition points.

	CO_2_ (ppm), median (IQR)	tVOCs (ppb), median (IQR)	PM1 (µg/m^3^), median (IQR)	PM2.5 (µg/m^3^), median (IQR)	PM10 (µg/m^3^), median (IQR)	RR (breaths/min), median (IQR)	PR (beats/min), median (IQR)
A	1010.4 (214.2)	586.1 (207.5)	8.4 (7.8)	9.9 (8.6)	10.4 (8.9)	24.0 (16.9)	86.7 (13.1)
B	615.9 (56.3)	167.7 (215.0)	22.7 (22.8)	24.2 (16.0)	24.6 (16.9)	17.1 (16.6)	98.8 (19.2)
C	628.7 (73.5)	161.8 (254.2)	19.7 (23.3)	23.0 (17.5)	23.3 (17.3)	14.3 (9.67)	98.1 (18.6)
D	619.4 (74.7)	160.4 (259.9)	21.5 (24.8)	21.7 (16.9)	22.0 (18.9)	14.6 (9.6)	97.7 (19.0)
E	628.5 (72.0)	86.1 (134.7)	20.7 (23.5)	23.3 (21.9)	23.7 (21.6)	14.3 (9.7)	105.1 (21.0)
F	626.6 (64.6)	134.5 (319.6)	21.7 (20.2)	21.8 (17.0)	22.1 (17.1)	11.1 (8.6)	99.0 (21.5)
G	650.0 (79.1)	205.8 (440.0)	24.4 (17.8)	26.2 (15.1)	26.6 (15.1)	11.3 (9.6)	101.0 (25.0)

In addition to the significantly different results between indoor and outdoor acquisition points, other statistically significant differences were detected between the distributions. For CO_2_, the acquisitions in point G, outside the laboratory in the parking lot near a low-traffic street, differ with a *P*<.05 from all the other acquisition points. The great variability of the indoor CO_2_ concentration, assessed visually in Figure 1, was confirmed also by a quantitative analysis, showing a wider IQR in the indoor acquisition of all participants with respect to all the other outdoor acquisitions. For what concerns tVOCs, point E near the train station, which is highly ventilated, shows significantly lower values with respect to point G (*P*<.001). PM concentration was significantly higher in point G (*P*<.001 for all PMs) with respect to the gas station (B), the park (C), and the intersection with traffic lights (D).

About the comparison between morning and afternoon acquisitions, significantly higher values of concentration were found in the afternoon (*P*<.001) for all the pollutants except CO_2_, which was higher in the morning (*P*<.001).

Regarding physiological parameters at various acquisition points, several noteworthy observations emerge.

The median RR at the initial acquisition point (A) was notably higher compared with all others, exhibiting a significant difference from points C, E, F, and G (with *P* values respectively *P*_AC_=.002, *P*_AE_=.001, *P*_AF_<.001, *P*_AG_=.001). In points C, D, and E, RR values are slightly higher than those in points F and G, although not significantly except for the couple CF (*P*_CF_=.049, *P*_CG_=.33, *P*_DF_=.26, *P*_DG_=.30, *P*_EF_=.647, *P*_EG_=.94).

Turning our attention to PR data, a remarkable disparity exists between point A and all other acquisition points (*P*<.001). Both RR and PR values showed great variability among participants, confirmed also by the Kruskal-Wallis one-way ANOVA on Ranks (*P*<.001 in both cases).

### Inhaled Dose

A participant-specific comparison of PAPE to PM2.5 computed with tabulated data for $V\prime_{m}$ of an activity level of 3 (13.26 L/min for women and 15.14 L/min for men) and ARPA daily concentration values (indicated by the subscript “STD”), and the assessment derived from the wearable system (indicated by the subscript “NEW”), is reported in Table 3.

**Table 3.** Particulate matter 2.5 (PM2.5) inhaled dose evaluated with the standard method (STD) and with the wearable system (NEW).

P#	*V’m*^_NEW_^ (L/min), median (IQR)	*V’m*_STD_ (L/min)	C^a^_NEW_ (μg/m^3^), median (IQR)	C_STD_ (μg/m^3^)	ID^b^_NEW_ (μg)	ID_STD_ (μg)
P01	20.24 (7.96)	13.26	36.52 (5.05)	17	24.93	7.89
P02	13.64 (6.64)	15.14	9.84 (9.88)	19	5.59	10.07
P03	20.35 (6.1)	15.14	20.77 (5.73)	19	15.18	10.07
P04	14.43 (4.09)	13.26	20.87 (4.36)	28	10.41	12.99
P05	24.75 (13.23)	15.14	31.07 (6.27)	28	31.41	14.84
P06	15.71 (9.14)	13.26	19.35 (7.4)	27	12.33	12.53
P07	23.32 (14.97)	15.14	22.27 (6.72)	27	23.81	14.31
P08	20.05 (12.86)	15.14	1.24 (0.96)	7	1.07	3.71
P09	21.31 (10.99)	15.14	37.04 (8.22)	29	26.81	15.37
P10	14.77 (7.14)	15.14	44.91 (7.24)	29	24.32	15.37
P11	18.82 (7.84)	15.14	12.51 (4.36)	30	9.34	15.90
P12	18.19 (6.71)	13.26	26.73 (8.9)	30	16.00	13.92
P13	22.73 (5.71)	15.14	20.14 (7.65)	28	14.83	14.84
P14	30.9 (12.45)	15.14	14.06 (10.2)	28	16.11	14.84
P15	8.72 (3.73)	13.26	9.7 (4.05)	20	3.06	9.28
P16	16.3 (8.44)	15.14	4.6 (1.6)	21	3.11	11.13
P17	15.06 (7.12)	13.26	11.77 (4.29)	21	6.32	9.75
P18	18.2 (5.27)	15.14	46.24 (6.84)	40	26.73	21.20
P19	26.11 (11.68)	15.14	37.06 (6.48)	36	31.92	19.08
P20	16.9 (5.61)	15.14	39.64 (9.05)	36	22.11	19.08

a C: concentration.

b ID: inhaled dose.

Computed $V\prime_{m}$ has a great variability among the different participants as highlighted by median values, but also intrasubject variability as can be observed analyzing the reported IQRs. It can be observed that the IQR values of C_NEW_ are very high, reaching a maximum of 10.2 μg/m^3^. This proves the great spatial and temporal variability of PM2.5 along the 4.5 km path and the ability of the developed wearable environmental monitor to account for that.

**Figure 6.**
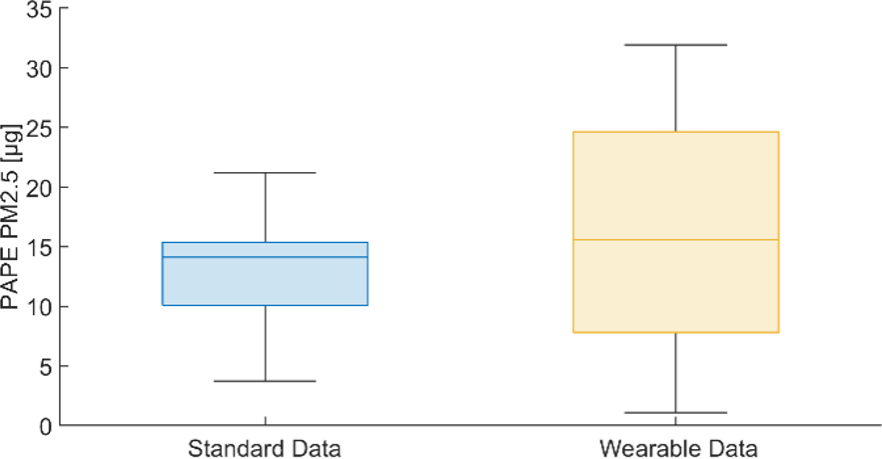
Boxplots representing PAPE to PM2.5 computed with standard data, which are ARPA values for pollutant’s concentration and tabulated values for minute ventilation, and with wearable data detected by the proposed wearable system. ARPA: Agenzia Regionale per la Protezione Ambientale; PAPE: personal air pollution exposure; PM: particulate matter.

It is calculated that using ARPA values of concentration and tabulated values of minute ventilation led to a mean PAPE to PM2.5 (ID_STD_ in Table 3) of 13.31 μg with a SD of 4.16 μg, whereas the PAPE obtained with our BSN (ID_NEW_ in Table 3) had a mean of 16.27 μg and a SD of 9.78 μg. They are strongly positively correlated (r=0.665, P<.001), indicating that when the dose estimated from wearable devices increases, the dose calculated from standard data also tends to increase; similarly, when there is a decrease in one measure, the other decreases as well. This alignment suggests a consistent relationship between the 2 methods, with changes in wearable-derived data generally reflecting those in the standard measurements, however, the method with wearable data exhibits higher variability in the estimations. The paired *t* test applied to the 2 normal distributions reveals that on average, the PAPE to PM2.5 with standard data underestimates by 2.96 μg the PAPE evaluated with recorded data coming from the proposed wearable system (22.3% difference; 95% CI −6.55 to 0.63, *P*=.05). Boxplots comparing the PAPE of PM2.5 are reported in Figure 6. In Table 4 the medians and IQRs of aggregated data of PAPE for the different acquisition points for CO_2_, VOC, PM1, PM2.5, and PM10 are reported.

**Table 4.** Personal air pollution exposure of CO_2_, total volatile organic compounds (tVOCs), particulate matter (PM) 1, PM2.5, and PM10 in the 7 acquisition points.

	CO_2_ (pp•mm^3^), median (IQR)	tVOCs (pp•bm^3^), median (IQR)	PM1 (µg), median (IQR)	PM2.5 (µg), median (IQR)	PM10 (µg), median (IQR)
A	108.3 (59.3)	108.3 (59.3)	0.7 (1.3)	0.8 (1.4)	0.9 (1.5)
B	69.9 (52.0)	69.9 (52.0)	1.8 (3.1)	2.1 (2.9)	2.4 (3.2)
C	80.1 (22.5)	80.1 (22.5)	2.5 (2.6)	2.3 (2.5)	2.6 (2.6)
D	65.8 (18.7)	65.8 (18.7)	2.4 (3.1)	2.3 (2.9)	2.5 (3.2)
E	72.2 (35.0)	72.2 (35.0)	2.5 (3.8)	2.5 (3.5)	2.7 (3.8)
F	67.3 (20.0)	67.3 (20.0)	2.6 (1.9)	2.1 (1.8)	2.4 (1.9)
G	71.2 (20.0)	71.2 (20.02)	2.5 (2.5)	2.2 (2.5)	2.4 (2.8)

The Friedman test resulted in a significant difference in PAPE between acquisition points for all the pollutants, with a *P*<.001. The post-hoc analysis on PAPE, with the Bonferroni correction, revealed that the only point that is statistically different from all the others is point A, which was expected since it is the only indoor location of the experimental campaign. Some considerations, even though not statistically significant, can be added. PAPE to CO_2_ is higher (*P*=.12) in point C (park) with respect to point F (small park with ARPA station). The situation is analogous for tVOCs (*P*=.10), with the additional factor that trees contribute to their emissions. Considering PAPE to PM near the station (E), higher values with respect to the other points were reached, even if statistically significant only compared with point C (*P*_BE_=.88, *P*_CE_=.03, *P*_DE_=.19, *P*_EF_=.58, *P*_EG_=.91).

### Questionnaire Results

**Figure 7.**
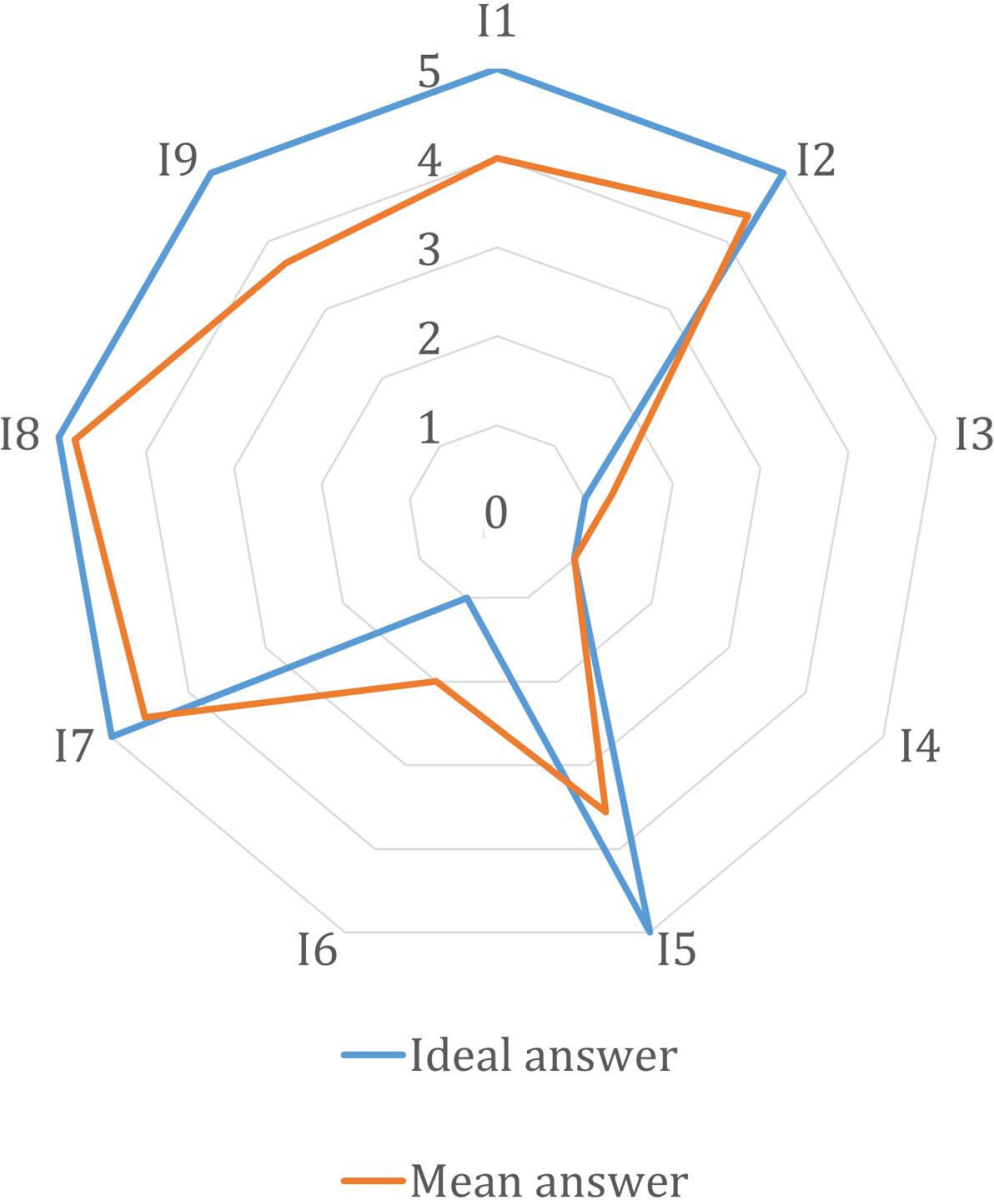
Radar plot of questionnaire results. Ideal scores are indicated with the blue line while in orange mean scores collected during the campaign.

The results of the questionnaire are presented in the radar plot in Figure 7, which displays both the collected scores and the optimal/ideal scores for each item. The lowest scores are obtained for statements regarding wearability over a long period and the functionality of the hardware design. Despite these concerns, high scores were obtained for the question addressing the comfort of the fixing method (I3 in Table 1). Additionally, the way data are presented in the accompanying application was appreciated, as demonstrated in I8.

## Discussion

### Main Results

This study aimed to determine the feasibility of using the proposed BSN for estimating inhaled pollutant doses in real-life settings. Additionally, it sought to assess whether the system could detect differences in pollution levels between morning and afternoon, evaluate variations across predefined locations with different pollution sources regardless of the time of day, and examine the suitability of the BSN for both indoor and outdoor measurements. The study also compared the system’s data with those from an ARPA fixed station and assessed the overall usability and user acceptance of the proposed solution.

The feasibility of using the proposed BSN for estimating the inhaled dose of pollutants in real-life settings is supported by the lightweight of the devices and their complete wearability. For what concerns the environmental monitor, the sensors chosen are specifically suited for high temporal resolution sampling, making the system well-equipped to track the substantial spatial and temporal variability of air pollution. This is particularly relevant given that pollutant concentrations can vary significantly over small spatial scales (<100 m) and short time periods due to emission dynamics, such as traffic peaks during rush hours. As highlighted in the literature, fixed monitoring stations may fail to capture these variations adequately, reinforcing the need for a high-resolution mobile system [7]. For comparison, commercial environmental monitoring instruments typically offer a measurement every minute at best, and more commonly, one sample every 5 minutes. Our device, which samples environmental parameters nearly 3 times faster than the fastest commercial options, is thus well-suited to capture the detailed spatial and temporal information required in environments like those tested. Moreover, the system includes units capable of synchronously retrieving RR and PR already aligned with the pollution data [26]. These units were already tested both in static [22] and dynamic conditions [27] in previous works. The synchronized data collection of the system allows for an accurate evaluation of the inhaled dose, calculated as the product of pollutant concentration and $V\prime_{m}$. Furthermore, the smartphone app has been thoroughly tested and performed reliably throughout the different tests. Therefore, the system is well-suited for estimating the inhaled dose of pollutants in real-life settings. It is worth noting that, although the sample size was determined by practical considerations, it also aligned with the sample sizes typically used in comparable studies focused on wearable prototypes or exposure assessment [22,28,29].

The system demonstrated its capability to study differences in pollution levels between morning and afternoon. CO_2_ was found to be higher during morning acquisitions with respect to afternoon acquisitions on the same day, both excluding and including the indoor environment in the analysis. This result is also confirmed in the literature. In fact, outdoor concentrations of CO_2_ exhibit daily and diurnal variations, with fluctuations ranging from 50 to 100 ppm in large urban centers, as was observed in a study in Baltimore [30]. CO_2_ concentrations tend to be lower in the afternoon and higher in the morning. Instead, PM concentrations were statistically higher in the afternoon compared with morning measurements. This trend aligns with the influence of traffic, which significantly elevates PM levels during peak hours such as lunchtime, coinciding with school dismissal and workers’ breaks.

The BSN effectively evaluated differences at predefined points along a walking path, each with potentially distinct pollution sources, regardless of the time of day. During the study, substantial fluctuations in PM were observed within a single data acquisition session, underscoring the limitations of stationary monitoring stations in capturing nuanced air quality dynamics. Indeed, over a short 4.5 km walk, individuals encounter diverse pollution levels, challenging the notion of a single daily concentration value as representative of actual exposure. For instance, in a low-traffic street in a parking lot (acquisition point G), PM levels were unexpectedly high, surpassing those at more conventionally polluted locations such as intersections with traffic lights. This elevated pollution was likely due to the architectural configuration around the parking lot, which impeded air recirculation, emphasizing the value of having a device that provides objective measurements rather than relying on subjective impressions. Physiological parameters further illustrate the system’s effectiveness in individually evaluating PAPE. At the initial acquisition point A indoors, participants showed a significantly higher RR, likely due to heightened anxiety due to the start of the experimental acquisition or the high CO_2_ concentration in the room. Currently, the system cannot distinguish between these factors because additional sensors, such as those measuring galvanic skin response, are needed to properly assess the participants’ stress levels. However, the system shows potential for future developments in this area. Furthermore, the transition from point A to point B, which involved ascending a flight of stairs, contributed to the observed higher, even if not significant, RR at point B with respect to the other acquisition points. RR further decreased at subsequent points, particularly where participants were seated, such as at points F and G. PR data showed lower values at point A, with consistent increases as participants moved and engaged in brisk walking, reflecting expected physiological responses. Additionally, variations in CO_2_ and tVOCs levels were observed across different points. Point C, a park with dense tree cover, had significantly higher CO_2_ and tVOCs readings compared with point F, a smaller park with less tree coverage. Studies suggest that VOCs from trees may have beneficial effects, such as enhancing cognitive functions and reducing mental fatigue [31]. Consequently, understanding whether a sensor can detect tree-derived VOCs—and at what concentrations—is essential for making informed decisions about exposure and potential benefits. For PM, the highest IQR was recorded at point E, near a train station, influenced by factors such as nearby taxis and varying traffic conditions. This variability, along with differences in physiological responses, underscores the importance of the BSN’s ability to provide nuanced data across various environments. Overall, the system’s capacity to detect and analyze these spatial and temporal variations confirms its effectiveness in evaluating pollution and exposure differences at multiple predefined points along a walking path.

The system has proven to be suitable for both indoor and outdoor measurements. Indoor measurements indicated significantly higher concentrations of CO_2_ and tVOCs, with considerable variability observed even within short, 5-minute intervals. This variability was particularly notable among different participants and can be attributed to varying conditions of air recirculation and fluctuations in the number of individuals in the room. In contrast, PM concentrations were generally lower indoors, consistently with expectations. Outdoors, the system detected notable variability in PM levels across different days and even within the same day, highlighting its capability to capture dynamic changes in air quality. This ability to effectively monitor and account for fluctuations in both indoor and outdoor environments confirms the system’s versatility and suitability for comprehensive air quality assessment. Given that urban populations spend most of their time indoors, where pollutant concentrations often exceed outdoor levels, the ability to monitor both environments is essential for accurately assessing personal exposure [7,32].

The data obtained from the new device show notable variability when compared with values reported by the ARPA fixed station. The new device typically recorded lower pollutant concentrations compared to the ARPA mean values. This discrepancy is primarily due to the experimental protocol, which intentionally avoided peak traffic hours, resulting in data collection during periods of lower pollutant concentrations. This highlights the limitations in time resolution of fixed stations, which may not fully capture the real exposure levels experienced by individuals. Moreover, fixed monitoring stations are often limited in their spatial representativeness, as air pollution levels can vary significantly even over small distances due to local emission sources, urban morphology, and meteorological conditions. The presence of street canyons, for example, can lead to pollutant accumulation, while open areas may experience greater dispersion. Consequently, a mobile system such as the one used in this study provides a more accurate representation of personal exposure by capturing both spatial and temporal variations that fixed stations may not be able to detect [7].

Additionally, PR and RR varied significantly among different participants and even in the same participants at different acquisition points. This variability was reflected in $V\prime_{m}$ data, which were computed using a model based on these physiological measurements. Unlike previous studies that assign standard $V_{m}^{\prime }$ values based on activity type [33], our approach accounts for individual physiological variations [34], improving the accuracy of inhaled pollutant dose estimation [8,32]. This individualized evaluation of inhaled dose is especially important, as shown in studies of athletes, where factors such as physical activity and ventilation rate significantly impact exposure levels, emphasizing the need for personalized assessment to better reflect real exposure [35]. Exemplary comparison of inhaled doses of PM2.5—calculated as the product of $V\prime_{m}$ and pollutant concentrations—revealed an average overestimation of 2.96 µg with the new wearable system compared with ARPA data and standard tables for $V\prime_{m}$, and also a higher variability in the measurements obtained from the BSN. These observations highlight the system’s ability to capture fine temporal and spatial variations in air quality and physiological parameters, demonstrating its potential for more accurate real-time monitoring compared with traditional fixed stations combined with tabulated data.

The usability of the proposed system was positively received by the tested population. The results from usability satisfaction questionnaires indicate an overall favorable perception of the system. Participants found it acceptable and expressed strong interest in its functionality. This positive feedback underscores the system’s practicality and user appeal and indicates its potential for broader application and adoption.

### Limitations

A limitation of the work is that physiological data were collected only in static positions, while the environmental monitor continuously detects pollution values. In fact, to properly assess PAPE during the testing session, also dynamic acquisitions with physiological parameters need to be performed, and future implementations should account for that. However, the collected dataset was enough to demonstrate significant variability in $V\prime_{m}$ among different participants, and also for the same participant. Future research should also include activities other than walking, such as cycling to enhance a wider range of minute ventilation values. Additionally, incorporating simultaneous measurements of 2 or more individuals walking the same route could provide further insights into individual variations in exposure to micro-environmental pollution and physiological responses. This approach could be considered for future experimental trials to better highlight the need for personalized environmental exposure and health impact assessments.

In reference to the usability satisfaction questionnaires, some limitations were evident. Wearability over prolonged durations and device functionality emerged as pivotal aspects, emphasizing the necessity for a more compact and lighter device. It is worth noting that the device was consistently worn at the level of the wrist during all acquisitions. However, alternative configurations, such as attaching it to a belt or to an armband, can be implemented for enhanced comfort. Despite differences in pollution levels observed over distances of several meters, the relatively small distance between the mouth or nose and the device in these configurations suggests that any of these placements could accurately measure pollutant exposure and reliably support PAPE assessment.

### Conclusions

Traditional air pollution exposure estimation methods fail to account for the dynamic variability in air pollution both indoors and outdoors, as well as the variations in minute ventilation among individuals. To overcome such limitations, our study introduces a novel wearable PAPE evaluation system consisting of a BSN collecting both physiological and environmental parameters, as concentrations of pollutants that have a known adverse effect on human health. Statistically significant differences in pollutant concentration were found between morning and afternoon but also between acquisition points in the same test session.

To estimate $V\prime_{m}$, we used a pre-existing model described in the literature. The PAPE was then computed by integrating over time the product of $V\prime_{m}$ and pollutant concentrations sampled by the developed system. As an example, PAPE to PM2.5, evaluated with fixed station’s concentration and standard values of $V\prime_{m}$ for the 20 participants, was significantly lower with respect to PAPE obtained with the proposed wearable system. Despite the study’s participants pool was limited, it effectively highlighted the variability in $V\prime_{m}$ among individuals and fluctuations in pollutant concentration.

Future improvements involve incorporating an active air recirculation system to mitigate micro-environmental effects inside the device’s 3D-printed case over temperature and humidity and advancing device miniaturization.

In conclusion, this system demonstrates high spatial and temporal resolution, positioning it as a valuable tool that may supplement epidemiological studies and empower the public with data to enhance pollution awareness and acceptability towards policies aimed at reducing air pollution.

## Abbreviations

ARPA: Agenzia Regionale per la Protezione Ambientale
BSN: body sensor network
HR: heart rate
IMU: inertial measurement unit
PAPE: personal air pollution exposure
PM: particulate matter
PR: pulse rate
RR: respiratory rate
tVOC: total volatile organic compound
VOC: volatile organic compound
